# Handgrip strength weakness and asymmetry together are associated with cardiovascular outcomes in older outpatients: A prospective cohort study

**DOI:** 10.1111/ggi.14451

**Published:** 2022-08-05

**Authors:** Siyang Lin, Fang Wang, Yanjie Huang, Yin Yuan, Feng Huang, Pengli Zhu

**Affiliations:** ^1^ Shengli Clinical Medical College of Fujian Medical University Fuzhou China; ^2^ Department of Geriatric Medicine Fujian Provincial Hospital Fuzhou China; ^3^ Fujian Health College Fuzhou China; ^4^ Nursing School of Fujian Medical University Fuzhou China; ^5^ Fujian Provincial Center for Geriatrics Fuzhou China; ^6^ Fujian Provincial Key Laboratory of Geriatrics Fuzhou China

**Keywords:** cardiovascular outcomes, handgrip strength asymmetry, handgrip strength weakness, older outpatients

## Abstract

**Aim:**

The evaluations of handgrip strength (HGS) weakness and asymmetry have implications for the comprehensive geriatric assessment. The aim of this study was to investigate the association of HGS weakness and asymmetry on cardiovascular outcomes in older outpatients.

**Methods:**

This was a prospective observational cohort study of 364 Geriatrics outpatients aged ≥60 years, in which all participants carried out HGS tests at baseline. Patients with HGS <28 kg for men and <18 kg for women were diagnosed as HGS weakness, and HGS ratio <0.90 or >1.10 were diagnosed as HGS asymmetry. Primary outcomes defined as the major adverse cardiovascular event and composite end‐points were assessed during a 21‐month median follow‐up.

**Results:**

Among 364 participants, 155 (42.6%) showed HGS weakness, and 160 (44.0%) showed HGS asymmetry. HGS weakness was associated with major adverse cardiovascular events (HR 2.76, 95% CI 1.22–6.27) and composite end‐points (HR 2.84, 95% CI 1.40–5.77). However, no significant correlation between HGS asymmetry and cardiovascular outcomes was observed. Compared with the normal and symmetric HGS group, older adults with HGS weakness and asymmetry together had a higher risk of major adverse cardiovascular events (HR 5.23, 95% CI 1.56–17.54) and composite end‐points (HR 4.00, 95% CI 1.56–10.28).

**Conclusions:**

HGS weakness and asymmetry together might increase the risk of cardiovascular outcomes in older outpatients. HGS asymmetry offers complementary information to HGS weakness when making a comprehensive assessment of HGS. **Geriatr Gerontol Int 2022; 22: 759–765**.

## Graphical abstract

Handgrip strength (HGS) weakness and asymmetry together might increase the risk of cardiovascular outcomes in older outpatients. HGS asymmetry offers complementary information to HGS weakness when making a comprehensive assessment of HGS.

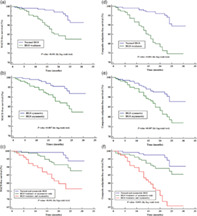

## Introduction

Handgrip strength (HGS) is a pragmatic measure of physical function for older people, as one of the diagnostic criteria of frailty and sarcopenia, which are common syndromes in the older population. HGS is also considered as an assessment of the locomotor dimension in intrinsic capacity for older adults. Several studies have shown that HGS weakness was associated with increased all‐cause mortality and death rates as a result of cardiovascular diseases.^1^, ^2^, ^3^ For instance, Leong *et al*. concluded that a decrease in HGS was positively associated with all‐cause mortality, myocardial infarction, stroke and cardiovascular morbidity in the Prospective Urban Rural Epidemiology (PURE) study.^4^ In addition, a 4‐year longitudinal study that included 3018 Chinese community‐dwelling older adults confirmed that HGS decreased with age.^5^ Timely evaluation of HGS might be helpful in identifying individuals at increased risk for adverse cardiovascular outcomes and premature mortality, especially for older adults.

However, HGS measurements mainly focused on HGS weakness only. Maximal HGS value was usually reported in the HGS test, whereas clinicians rarely took HGS asymmetry between left and right hands seriously. In recent 2 years, McGrath *et al*. carried out in‐depth research on HGS asymmetry and adverse events in older adults, such as falls, functional limitations, cognitive disorders and mortality.^6^, ^7^, ^8^, ^9^, ^10^ There is uncertainty whether HGS asymmetry is associated with cardiovascular outcomes, which is of great importance in assessing the health of the older population.

Atherosclerosis might contribute to the development of HGS weakness.^11^ Meanwhile, HGS weakness provides a valid marker of muscle strength weakness,^12^ nutritional status.^13^ These potential mechanisms can be used as a bridge to correlate HGS weakness with cardiovascular outcomes. Furthermore, HGS asymmetry might indicate deficient brain hemisphere activation and impairment of the neuromuscular system.^14^ Adding HGS asymmetry to the basis of HGS weakness assessment might enhance risk prediction for cardiovascular outcomes in older adults.

We hypothesized that older adults with HGS weakness and asymmetry together might have a higher risk of cardiovascular outcomes. Therefore, the present study aimed to analyze the association of HGS weakness and asymmetry on cardiovascular outcomes in older outpatients.

## Methods

### Study population

The present prospective cohort study was carried out in Fujian Provincial Hospital from December 2015 to July 2017. A total of 408 participants were recruited from the Geriatrics outpatients. The inclusion criteria were as follows: (i) aged ≥60 years; and (ii) written informed consent. Participants who met one of the following criteria were excluded: (i) acute, critical or terminal stages of various diseases; (ii) diagnosed with malignant tumors; (iii) severe neurological or psychiatric disorders; (iv) disability or immobility due to severe osteoarthritis or neuromuscular disease; (v) a history of myocardial infarction and stroke; (vi) New York Heart Association (NYHA) class III–IV; and (vii) hospitalization with unstable angina or heart failure in the past 6 months. The follow‐up ended in January 2019, and 44 participants were lost to follow‐up. A total of 364 participants were included in the final analysis. Approval for the study was obtained from the Fujian Provincial Hospital research ethics committee (KY2015‐09‐01).

### HGS weakness and asymmetry measurement

HGS was assessed using a handheld hydraulic dynamometer (Jamar, Anaheim, CA, USA) in the sitting position with the forearm in the neutral position. There was a familiarization trial before the registered trials. Participants squeezed the dynamometer with the arm elbow bent to a 90° angle as hard as possible. The maximum reading of three trials for both hands was taken as HGS value (kg). Every between‐trial interval was more than 15 s to avoid muscle fatigue.^15^ According to the Asian Working Group for Sarcopenia 2019 consensus, HGS weakness was defined as an HGS of <28 kg in men and <18 kg in women.^16^ The highest recorded HGS from both hands were used to calculate the HGS ratio (dominant HGS (kg) / non‐dominant HGS (kg)).^17^ The “10% rule” was first proposed in the 1950s, indicating that the difference in HGS between dominant and non‐dominant hands was approximately 10%.^17^ Numerous studies associated with HGS asymmetry applied the definition of an HGS ratio >10%.^7^, ^8^, ^9^, ^10^, ^18^, ^19^, ^20^, ^21^ Accordingly, HGS asymmetry was considered as an HGS ratio <0.90 or >1.10, and HGS symmetry was classified as an HGS ratio between 0.90 and 1.10 in the present study.

### Follow up and outcomes

All participants were followed up routinely every 6 months by telephone to obtain their survival data and record the time‐to‐event end‐points. All records were reviewed by well‐trained staff. The death records were obtained from the participant's family and medical records. Cardiovascular diseases and causes of death were codified according to the 10th version of International Classification of Diseases (ICD).

Major adverse cardiovascular event (MACE) was defined as cardiac death (ICD‐10: 100–199), acute myocardial infarction (ICD‐10: I21), hospitalization for unstable angina (ICD‐10: I20), hospitalization for congestive heart failure (ICD‐10: I50) and acute stroke (ICD‐10: I60‐64, I67, I69). Composite end‐points were defined as all‐cause mortality, acute myocardial infarction (ICD‐10: I21), hospitalization for unstable angina (ICD‐10: I20), hospitalization for congestive heart failure (ICD‐10: I50) and acute stroke (ICD‐10: I60‐64, I67, I69). The end‐point of the study was the time to the first recorded adverse event.

### Covariates

Age and sex were verified using the participants' identification cards. The professional medical staff measured the height and weight of the participants. Body mass index was calculated as weight divided by height squared. Smoking and drinking status were self‐reported, divided into never and ever or current usage. The participants were asked if they had been diagnosed with cardiovascular diseases (hypertension, heart failure [HF] and coronary heart disease) or metabolic disorders (diabetes and hyperlipidemia) in medical institutions. The covariates were selected based on clinical relevance, which were recognized as risk factors for cardiovascular outcomes.

### Statistical analysis

Statistical analysis was carried out using Statistical Package for the Social Sciences, version 22.0 (IBM Corporation, Armonk, NY, USA). Figures were drawn using GraphPad Prism 8.0 (GraphPad Software, San Diego, CA, USA) and MedCalc (MedCalc Software, Ostend, Belgium). Continuous variables of the normal distribution are presented as the mean and standard deviation. Continuous variables of non‐normal distribution are presented as the median and interquartile range. Categorical variables are presented as frequencies and percentages (%). Homogeneity of variance test and normality analysis were carried out before comparing two groups. Independent samples *t*‐test or one‐way anova were used for continuous variables of normal distribution, and the non‐parametric test was used for continuous variables of non‐normal distribution. The differences in categorical variables were evaluated by the χ^2^‐test or Fisher's exact test.

Survival analysis was carried out using the Kaplan–Meier method and Cox proportional hazards regression analysis. Kaplan–Meier curves were constructed to evaluate the association of HGS weakness and HGS asymmetry at baseline on MACE or composite end‐points. The log‐rank statistic was calculated for each curve. Cox proportional hazard models were used to estimate hazard ratio (HRs) and 95% CIs for MACE and composite end‐points, comparing HGS weakness categories (the reference group: normal HGS), HGS asymmetry categories (the reference group: HGS symmetry), HGS weakness and asymmetry categories (grouped into normal and symmetric HGS [the reference group], HGS weakness or asymmetry only, HGS weakness and asymmetry), which were presented as three models. Model 1 was unadjusted for confounders. In Model 2, the analysis was adjusted for age and sex. Model 3 was adjusted as model 2 with body mass index, smoking, drinking, hypertension, diabetes, hyperlipidemia, HF and coronary heart disease. The hypothesis test was carried out by a two‐sided test, and statistical significance was set at *P* < 0.05.

## Results

After excluding 44 participants lost to follow‐up, 364 participants (mean 72.4 ± 8.3 years) were included in the study, nearly half (49.7%) of them were men. Table 1 shows the baseline characteristics of the study participants between the HGS symmetry and HGS asymmetry group. There were 155 (42.6%) older adults with HGS weakness, 160 (44.0%) with HGS asymmetry, and 78 (21.4%) with HGS weakness and asymmetry together. The incidence of cardiovascular outcomes during the follow‐up period, of which the median observation time was 21.0 months (interquartile range 18.5–26.4), is shown in Table S1. Of 364 participants, 35 (9.6%) older adults developed MACE and 50 (13.7%) presented composite end‐points.

**Table 1:** General characteristics of participants at baseline

Characteristics	Total (*n* = 364)	HGS symmetry (*n* = 204)	HGS asymmetry (*n* = 160)	*P*‐value
Age (years)	72.4 ± 8.3	70.7 ± 7.6	74.5 ± 8.8	<0.001
Men, *n* (%)	181 (49.7)	105 (51.5)	76 (47.5)	0.452
BMI (kg/m^2^)	23.8 ± 3.5	24.0 ± 3.4	23.5 ± 3.5	0.082
Smoking, *n* (%)	60 (16.5)	38 (18.6)	22 (13.8)	0.213
Drinking, *n* (%)	27 (7.4)	16 (7.8)	11 (6.9)	0.726
Hypertension, *n* (%)	249 (68.4)	150 (73.5)	99 (61.9)	0.018
Diabetes, *n* (%)	150 (41.2)	92 (45.1)	58 (36.3)	0.089
Hyperlipidemia, *n* (%)	96 (26.4)	61 (29.9)	35 (21.9)	0.085
HF, *n* (%)	25 (6.9)	12 (5.9)	13 (8.1)	0.401
CHD, *n* (%)	94 (25.8)	48 (23.5)	46 (28.7)	0.259
HGS weakness, *n* (%)	155 (42.6)	77 (37.7)	78 (48.8)	0.035
FBG	6.03 ± 1.66	6.04 ± 1.55	6.02 ± 1.79	0.571
TG (mmol/L)	1.30 (0.93, 1.81)	1.33 (0.90, 1.79)	1.24 (0.94, 1.82)	0.519
TC (mmol/L)	4.64 ± 1.15	4.78 ± 1.22	4.47 ± 1.04	0.021
LDL‐C (mmol/L)	2.86 ± 0.89	2.94 ± 0.89	2.76 ± 0.88	0.059
HDL‐C (mmol/L)	1.30 ± 0.50	1.35 ± 0.58	1.23 ± 0.37	0.068

BMI, body mass index; HF, heart failure; CHD, coronary heart disease; FBG, fasting blood glucose; HGS, handgrip strength; LDL‐C, low‐density lipoprotein protein cholesterol; HDL‐C, high‐density lipoprotein cholesterol; TC, total cholesterol; TG, triglycerides.

**Figure 1:**
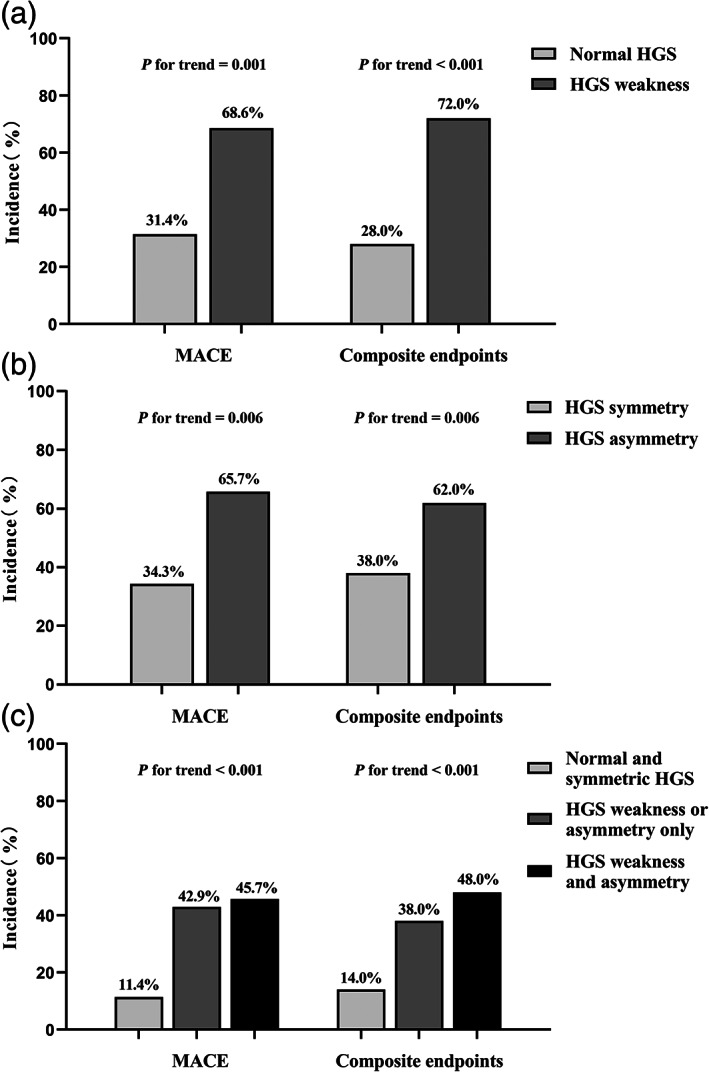
Incidence of cardiovascular outcomes in older outpatients by different handgrip strength weakness and handgrip strength asymmetry groups. HGS, handgrip strength; MACE, major adverse cardiovascular event

Participants with HGS weakness developed more cardiovascular outcomes with a higher incidence of MACE (*P* for trend = 0.001) and composite end‐points (*P* for trend <0.001) than the normal HGS group. Likewise, the incidence of MACE (*P* for trend = 0.006) and composite end‐points (*P* for trend = 0.006) in the HGS asymmetry group were higher than that in the HGS symmetry group. Combined with HGS weakness and HGS asymmetry, older adults were classified into three groups: normal and symmetric HGS group (*n* = 127), HGS weakness or asymmetry only (*n* = 159), and HGS weakness and asymmetry group (*n* = 78). Participants with HGS weakness and asymmetry together had the highest incidence of MACE (*P* for trend <0.001) and composite end‐points (*P* for trend <0.001) between the three groups (Fig. 1).

**Figure 2:**
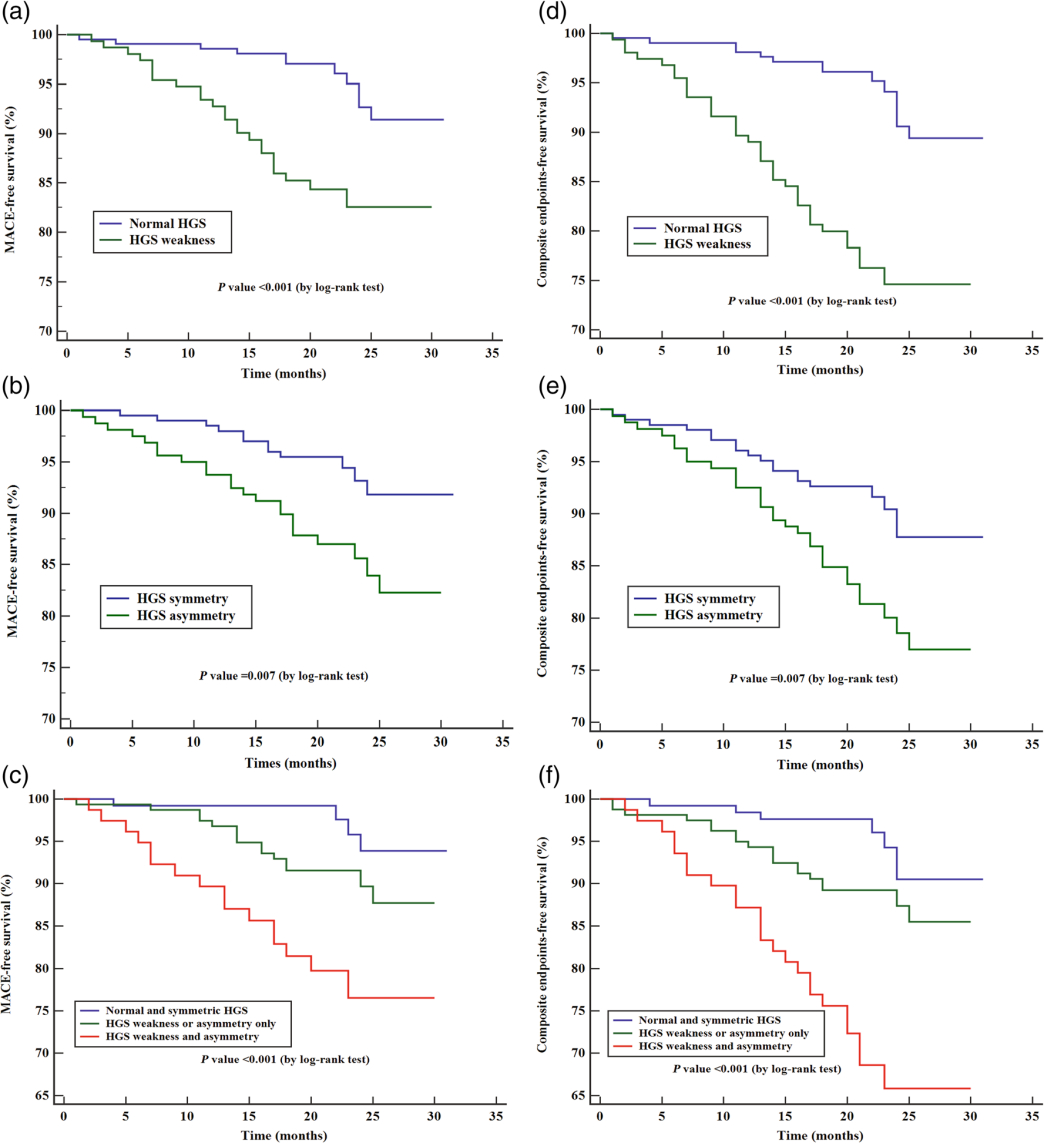
Kaplan–Meier survival curve of cardiovascular outcomes in older outpatients by different handgrip strength weakness and handgrip strength asymmetry groups. HGS, handgrip strength. Major adverse cardiovascular event (MACE): a composite of cardiac death, acute myocardial infarction, unstable hospitalization for unstable angina, hospitalization for congestive heart failure and acute stroke. Composite end‐points: a composite of all‐cause mortality, acute myocardial infarction, hospitalization for unstable angina, hospitalization for congestive heart failure and acute stroke.

In the survival analysis, MACE‐free survival rates and composite end‐points survival rates of the HGS weakness groups (*P* < 0.001) and HGS asymmetry groups (*P* = 0.007) were statistically significant according to the log‐rank test of the Kaplan–Meier curve. Significant differences also existed among the normal and symmetric HGS group, HGS weakness or asymmetry only group, and HGS weakness and asymmetry group (*P* < 0.001; Fig. 2).

Cox regression analysis was carried out to assess the association of HGS weakness and HGS asymmetry separately on cardiovascular outcomes in older outpatients (Table 2). The HR in the fully adjusted (model 3) model of HGS weakness for predicting MACE was 2.76 (95% CI 1.22–6.27, *P* = 0.015) and composite endpoints was 2.84 (95% CI 1.40–5.77, *P* = 0.004). However, HGS asymmetry could not predict MACE (HR 1.94, 95% CI 0.92–4.12, *P* = 0.083) and composite end‐points (HR 1.71, 95% CI 0.92–3.19, *P* = 0.089).

**Table 2:** Association of handgrip strength weakness and handgrip strength asymmetry separately on cardiovascular outcomes in older outpatients

	HGS weakness		HGS asymmetry	
	HR (95% CI)	*P* value	HR (95% CI)	*P*‐value
MACE				
Model 1	3.39 (1.66–6.92)	0.001	2.51 (1.25–5.04)	0.010
Model 2	2.26 (1.05–4.89)	0.038	2.11 (1.04–4.29)	0.040
Model 3	2.76 (1.22–6.27)	0.015	1.94 (0.92–4.12)	0.083
Composite end‐points				
Model 1	3.97 (2.14–7.36)	<0.001	2.14 (1.21–3.78)	0.009
Model 2	2.59 (1.33–5.03)	0.005	1.80 (1.01–3.21)	0.048
Model 3	2.84 (1.40–5.77)	0.004	1.71 (0.92–3.19)	0.089

Composite endpoints: a composite of all‐cause mortality, acute myocardial infarction, hospitalization for unstable angina, hospitalization for congestive heart failure, and acute stroke. Model 1: unadjusted. Model 2: adjusted for age and sex. Model 3: adjusted as model 2 with body mass index, smoking, drinking, hypertension, diabetes, hyperlipidemia, heart failure and coronary heart disease.

HGS, handgrip strength; MACE, major adverse cardiovascular event (a composite of cardiac death, acute myocardial infarction, hospitalization for unstable angina, hospitalization for congestive heart failure and acute stroke).

In Table 3, participants with HGS weakness and asymmetry had a 5.23 (95% CI 1.56–17.54) higher HR for MACE and a 4.00 (95% CI 1.56–10.28) higher HR for composite end‐points, whereas the HGS weakness or asymmetry only group had no statistical significance for predicting MACE and composite end‐points (*P* > 0.05).

**Table 3:** Association of handgrip strength weakness and asymmetry together on cardiovascular outcomes in elderly outpatients

	Normal and symmetric HGS (*n* = 127)		HGS weakness or asymmetry only (*n* = 159)		HGS weakness and asymmetry (*n* = 78)	
	HR (95% CI)	*P*‐value	HR (95% CI)	*P*‐value	HR (95% CI)	*P*‐value
MACE						
Model 1	1 (control)	–	3.21 (1.07–9.68)	0.038	7.56 (2.53–22.63)	<0.001
Model 2	1 (control)	–	2.86 (0.95–8.67)	0.063	4.74 (1.49–15.05)	0.008
Model 3	1 (control)	–	3.18 (0.99–10.17)	0.051	5.23 (1.56–17.54)	0.007
Composite end‐points						
Model 1	1 (control)	–	2.31 (0.97–5.49)	0.059	6.46 (2.78–15.00)	<0.001
Model 2	1 (control)	–	2.02 (0.85–4.83)	0.114	3.93 (1.60–9.61)	0.003
Model 3	1 (control)	–	1.94 (0.77–4.88)	0.157	4.00 (1.56–10.28)	0.004

HGS, handgrip strength; MACE, major adverse cardiovascular event (a composite of cardiac death, acute myocardial infarction, hospitalization for unstable angina, hospitalization for congestive heart failure, and acute stroke). Composite end‐points: a composite of all‐cause mortality, acute myocardial infarction, hospitalization for unstable angina, hospitalization for congestive heart failure, and acute stroke. Model 1: unadjusted. Model 2: adjusted for age and sex. Model 3: adjusted as Model 2 with body mass index, smoking, drinking, hypertension, diabetes, hyperlipidemia, heart failure and coronary heart disease.

## Discussion

The present study explored the association of HGS weakness and HGS asymmetry on cardiovascular outcomes represented by MACE and composite end‐points in older outpatients. The results showed that HGS weakness was associated with MACE and composite end‐points, whereas HGS asymmetry alone was not. Compared with the normal and symmetric group, older adults with HGS weakness and asymmetry together had a higher risk of MACE and composite end‐points, which showed the predictive value of HGS weakness and HGS asymmetry in combination for cardiovascular outcomes.

The epidemiological data of HGS asymmetry were limited, and there was also no unified diagnostic criterion for HGS asymmetry. The present study applied the “10% rule”, because it has been used for HGS asymmetry in most studies. In this study, the prevalence of HGS asymmetry was 44.0%, consistent with epidemiological information (44.3%) in older Americans for the same age range. Secondary analyses of data from the English Longitudinal Study of Ageing (ELSA) showed the prevalence of HGS asymmetry was 46.2% in the older population aged ≥50 years.^21^ A total of 15.9% of older Koreans were diagnosed with HGS asymmetry (20% rule) in a nationwide, population‐based, cross‐sectional study.^22^ To date, no studies have reported HGS asymmetry data in the Chinese older population.

The association between HGS weakness and cardiovascular outcomes was similar to previous studies. Some large epidemiological cohorts from different countries focused on HGS and cardiovascular outcomes. The PURE study carried out in 17 countries with a median follow‐up period of 4 years suggested that measurement of HGS was an effective risk‐stratifying method for all‐cause mortality, cardiac death and cardiovascular disease.^4^ In the prospective cohort study of half a million UK Biobank participants, HGS weakness was associated with all‐cause and cardiovascular mortality.^1^ The Korean Longitudinal Study of Aging (KLoSA) found that HGS was longitudinally related to the occurrence of cardiovascular diseases, such as heart disease (angina, myocardial infarction, congestive heart failure) and stroke.^3^ Another study from KLoSA showed that lower HGS was an independent predictor of all‐cause and cardiovascular mortality.^2^ Several meta‐analyses also confirmed the correlation between HGS and cardiac adverse events.^23^, ^24^ Rita Pavasini *et al*. carried out a meta‐analysis of patients with cardiac disorders (ischemic heart disease, HF, cardiomyopathies, valvulopathies, arrhythmias), which concluded that HGS emerged as a predictor of all‐cause death, cardiac death and hospital admission for HF. However, they did not find any relationship between HGS and the occurrence of cerebrovascular accidents or myocardial infarction.^23^

Arterial stiffness, physical activity and nutrition might mediate the association between HGS weakness and cardiovascular outcomes. First, a prospective study of older Dutch men in the community reported higher baseline carotid intima media thickness associated with low HGS after 4‐year follow up.^11^ In people with muscle strength weakness, chronic inflammation increases^25^ to reduce the bioavailability of nitric oxide, aggravate endothelial dysfunction, and accelerate atherosclerosis and arterial plaque formation by autocrine and paracrine mechanisms.^26^ Meanwhile, insufficient levels of physical activity influence muscle weakness, which in turn shows HGS weakness.^24^ In addition, HGS is a useful functional measure when added to a clinical nutrition assessment,^27^ and it can reflect the dietary intake in older adults.^28^ As a result of these factors, older people with HGS weakness are more likely to develop cardiovascular events.

In the present study, HGS asymmetry was not associated with MACE and composite end‐points after being fully adjusted in model 3, which showed that HGS asymmetry was not as effective as HGS weakness in predicting cardiovascular outcomes. One of the explanations might be the disuse of the non‐dominant limb.^29^ Lack of exercise in the non‐dominant hand generates a gap in HGS with the dominant hand. In contrast, HGS asymmetry can be explained by the asymmetry of the primary somatosensory cortex in each hemisphere of the brain and cerebellar‐related neurological dysfunction.^8^ The complex correlation between HGS asymmetry and the nervous system exactly supported that the aging people with HGS asymmetry might have a higher risk of falls and functional limitations discussed in several studies.^6^, ^9^, ^10^ Nevertheless, further research is required to identify whether HGS asymmetry can be an independent predictor for other adverse events, such as cardiovascular outcomes.

After consideration for both HGS weakness and asymmetry, an important conclusion can be obtained that HGS weakness and asymmetry together were associated with cardiovascular outcomes in older adults compared with those with normal and symmetric HGS. HGS asymmetry offers complementary information to HGS weakness when making a complete evaluation of HGS, implying the dysfunction of the neuromuscular system and cardiovascular system. The combination of HGS weakness and HGS asymmetry might improve the prediction ability of HGS assessment in cardiovascular outcomes, which have implications for comprehensive geriatric assessment. Implementing HGS asymmetry measurement in clinical practice merits greater attention, and more geriatricians and clinicians should target appropriate interventions for older patients with both HGS weakness and asymmetry.

The present study included some strengths that need to be acknowledged. Our study is one of the first studies adding HGS asymmetry to the assessment of HGS. Additionally, the study is the first to explore the longitudinal association of HGS weakness and asymmetry together on cardiovascular outcomes in older adults. The study also had some limitations that should be considered. First, the current sample size might not be sufficient, and the included population was geriatric outpatients rather than a community‐based older population. The results might not be considered relevant to the population at large. Second, the primary end‐point of this study was cardiovascular outcomes, and the baseline population did not fully exclude patients with coronary heart disease and HF. However, efforts were made to exclude patients with poor cardiac function and hospitalization for myocardial infarction and HF in the recent 6 months to reduce potential bias. Third, although we chose the “10% rule” as the threshold of HGS asymmetry, there still exist differences in HGS between hands. The underlying mechanism of HGS asymmetry needs to be confirmed in future biological or large longitudinal studies.

In conclusion, the present study identified the association of HGS weakness and asymmetry on cardiovascular outcomes. Older outpatients with HGS weakness and asymmetry together had a higher risk of cardiovascular outcomes. The findings suggested that HGS asymmetry in combination with HGS weakness could improve the predictive value of cardiovascular outcomes. It is necessary to carry out developed research on the mechanism and interventions of HGS asymmetry in the future.

## Disclosure statement

The authors declare no conflict of interest.

## Supporting information


**Table S1.** The incidence of major outcomes in older outpatients during the follow up.

## Data Availability

The data that support the findings of this study are available from the corresponding author upon reasonable request.

## References

[1] Celis‐Morales CA , Welsh P , Lyall DM *et al*. Associations of grip strength with cardiovascular, respiratory, and cancer outcomes and all cause mortality: prospective cohort study of half a million UKbiobank participants. BMJ 2018; 361: k1651. 29739772 10.1136/bmj.k1651 PMC5939721

[2] Kim GR , Sun J , Han M , Park S , Nam CM . Impact of handgrip strength on cardiovascular, cancer and all‐cause mortality in the Korean longitudinal study of ageing. BMJ Open 2019; 9: e027019. 10.1136/bmjopen-2018-027019 PMC6527975 31072857

[3] Jang SK , Kim JH , Lee Y . Effect of relative handgrip strength on cardiovascular disease among Korean adults aged 45 years and older: results from the Korean longitudinal study of aging (2006‐2016). Arch Gerontol Geriatr 2020; 86: 103937. 31574451 10.1016/j.archger.2019.103937

[4] Leong DP , Teo KK , Rangarajan S *et al*. Prognostic value of grip strength: findings from the prospective urban rural epidemiology (PURE) study. Lancet 2015; 386: 266–273. 25982160 10.1016/S0140-6736(14)62000-6

[5] Auyeung TW , Lee SW , Leung J , Kwok T , Woo J . Age‐associated decline of muscle mass, grip strength and gait speed: a 4‐year longitudinal study of 3018 community‐dwelling older Chinese. Geriatr Gerontol Int 2014; 14: 76–84. 24450564 10.1111/ggi.12213

[6] McGrath R , Clark BC , Cesari M , Johnson C , Jurivich DA . Handgrip strength asymmetry is associated with future falls in older Americans. Aging Clin Exp Res 2021; 33: 2461–2469. 33247424 10.1007/s40520-020-01757-z PMC8211412

[7] McGrath R , Cawthon PM , Cesari M , Al Snih S , Clark BC . Handgrip strength asymmetry and weakness are associated with lower cognitive function: A panel study. J Am Geriatr Soc 2020; 68: 2051–2058. 32473060 10.1111/jgs.16556

[8] McGrath R , Tomkinson GR , LaRoche DP , Vincent BM , Bond CW , Hackney KJ . Handgrip strength asymmetry and weakness may accelerate time to mortality in aging Americans. J Am Med Dir Assoc 2020; 21: 2003–2007.e1. 32611522 10.1016/j.jamda.2020.04.030

[9] Collins K , Johnson N , Klawitter L *et al*. Handgrip strength asymmetry and weakness are differentially associated with functional limitations in older Americans. Int J Environ Res Public Health 2020; 17: 3231. 10.3390/ijerph17093231 PMC7246814 32384713

[10] McGrath R , Vincent BM , Jurivich DA *et al*. Handgrip strength asymmetry and weakness together are associated with functional disability in aging Americans. J Gerontol, Ser A 2021; 76: 291–296. 10.1093/gerona/glaa100 32319511

[11] den Ouden ME , Schuurmans MJ , Arts IE *et al*. Atherosclerosis and physical functioning in older men, a longitudinal study. J Nutr Health Aging 2013; 17: 97–104.2329938710.1007/s12603-012-0424-2

[12] Bohannon RW , Magasi SR , Bubela DJ , Wang YC , Gershon RC . Grip and knee extension muscle strength reflect a common construct among adults. Muscle Nerve 2012; 46: 555–558. 22987697 10.1002/mus.23350 PMC3448119

[13] Norman K , Stobäus N , Gonzalez MC , Schulzke JD , Pirlich M . Hand grip strength: outcome predictor and marker of nutritional status. Clin Nutr 2011; 30: 135–142. 21035927 10.1016/j.clnu.2010.09.010

[14] Mitchell M , Martin BJ , Adamo DE . Upper limb asymmetry in the sense of effort is dependent on force level. Front Psychol 2017; 8: 643. 28491047 10.3389/fpsyg.2017.00643 PMC5405061

[15] Reijnierse EM , de Jong N , Trappenburg MC *et al*. Assessment of maximal handgrip strength: how many attempts are needed. J Cachexia Sarcopenia Muscle 2017; 8: 466–474. 28150387 10.1002/jcsm.12181 PMC5476859

[16] Chen LK , Woo J , Assantachai P *et al*. Asian working Group for Sarcopenia: 2019 consensus update on sarcopenia diagnosis and treatment. J Am Med Dir Assoc 2020; 21: 300–307.e2. 32033882 10.1016/j.jamda.2019.12.012

[17] Armstrong CA , Oldham JA . A comparison of dominant and non‐dominant hand strengths. J Hand Surg 1999; 24: 421–425. 10.1054/jhsb.1999.0236 10473148

[18] Chen Z , Ho M , Chau PH . Handgrip strength asymmetry is associated with the risk of neurodegenerative disorders among Chinese older adults. J Cachexia Sarcopenia Muscle 2022; 13: 1013–1023. 35178892 10.1002/jcsm.12933 PMC8977973

[19] Klawitter L , Vincent BM , Choi BJ *et al*. Handgrip strength asymmetry and weakness are associated with future morbidity accumulation in Americans. J Strength Cond Res 2022; 36: 106–112. 34941610 10.1519/JSC.0000000000004166

[20] Mahoney SJ , Hackney KJ , Jurivich DA , Dahl LJ , Johnson C , McGrath R . Handgrip strength asymmetry is associated with limitations in individual basic self‐care tasks. J Appl Gerontol 2022; 41: 450–454. 33356740 10.1177/0733464820982409

[21] Liu M , Liu S , Sun S , Tian H , Li S , Wu Y . Sex differences in the associations of handgrip strength and asymmetry with multimorbidity: evidence from the English longitudinal study of ageing. J Am Med Dir Assoc 2022; 23: 493–498.e1. 34389337 10.1016/j.jamda.2021.07.011

[22] Go YJ , Lee DC , Lee HJ . Association between handgrip strength asymmetry and falls in elderly Koreans: A nationwide population‐based cross‐sectional study. Arch Gerontol Geriatr 2021; 96: 104470. 34243024 10.1016/j.archger.2021.104470

[23] Pavasini R , Serenelli M , Celis‐Morales CA *et al*. Grip strength predicts cardiac adverse events in patients with cardiac disorders: an individual patient pooled meta‐analysis. Heart 2019; 105: 834–841. 30455175 10.1136/heartjnl-2018-313816

[24] Soysal P , Hurst C , Demurtas J *et al*. Handgrip strength and health outcomes: umbrella review of systematic reviews with meta‐analyses of observational studies. J Sport Health Sci 2021; 10: 290–295. 32565244 10.1016/j.jshs.2020.06.009 PMC8167328

[25] Strasser B , Keinrad M , Haber P , Schobersberger W . Efficacy of systematic endurance and resistance training on muscle strength and endurance performance in elderly adults – a randomized controlled trial. Wien Klin Wochenschr 2009; 121: 757–764. 20047114 10.1007/s00508-009-1273-9

[26] Guizoni DM , Dorighello GG , Oliveira HC , Delbin MA , Krieger MH , Davel AP . Aerobic exercise training protects against endothelial dysfunction by increasing nitric oxide and hydrogen peroxide production in LDL receptor‐deficient mice. J Transl Med 2016; 14: 213. 27435231 10.1186/s12967-016-0972-z PMC4950099

[27] McNicholl T , Dubin JA , Curtis L *et al*. Handgrip strength, but not 5‐meter walk, adds value to a clinical nutrition assessment. Nutr Clin Pract 2019; 34: 428–435. 30288776 10.1002/ncp.10198

[28] Tak YJ , Lee JG , Yi YH *et al*. Association of Handgrip Strength with dietary intake in the Korean population: findings based on the seventh Korea National Health and Nutrition Examination Survey (KNHANES VII‐1), 2016. Nutrients 2018; 10: 1180. 10.3390/nu10091180 PMC6165190 30154371

[29] Straight CR , Brady AO , Evans EM . Asymmetry in leg extension power impacts physical function in community‐dwelling older women. Menopause 2016; 23: 410–416. 26645817 10.1097/GME.0000000000000543

