# Supplementary material for: Handgrip strength weakness and asymmetry together are associated with cardiovascular outcomes in older outpatients: A prospective cohort study
Source: Geriatr Gerontol Int. 2022 Aug 5;22(9):759–65. doi: 10.1111/ggi.14451 (PMC9544274; doi:10.1111/ggi.14451)
Supplement: Supplementary file 1 — Table S1. The incidence of major outcomes in older outpatients during the follow up. [file GGI-22-759-s001.doc]

Table S1 The incidence of major outcomes in elderly outpatients during the follow-up

| **Outcomes** | **Total (n=364)** |
| --- | --- |
| Composite endpoints, n (%) | 50 (13.7) |
| All-cause mortality, n (%) | 16 (4.4) |
| MACE, n (%) | 35 (9.6) |
| Cardiac death, n (%) | 1 (0.3) |
| Acute myocardial infarction, n (%) | 7 (1.9) |
| Unstable angina, n (%) | 4 (1.1) |
| Congestive heart failure, n (%) | 18 (4.9) |
| Acute stroke, n (%) | 5 (1.4) |

Composite endpoints: a composite of all-cause mortality, acute myocardial infarction, hospitalization for unstable angina, hospitalization for congestive heart failure, and acute stroke. MACE: a composite of cardiac death, acute myocardial infarction, hospitalization for unstable angina, hospitalization for congestive heart failure, and acute stroke.
